# The value of pregnancy complication history for 10-year cardiovascular disease risk prediction in middle-aged women

**DOI:** 10.1007/s10654-018-0429-1

**Published:** 2018-07-30

**Authors:** Simon Timpka, Abigail Fraser, Tommy Schyman, Jennifer J. Stuart, Bjørn Olav Åsvold, Ingrid Mogren, Paul W. Franks, Janet W. Rich-Edwards

**Affiliations:** 1 Connors Center for Women’s Health and Gender Biology, Brigham and Women’s Hospital and Harvard Medical School, Boston, MA USA; 2 Genetic and Molecular Epidemiology Unit, Lund University Diabetes Centre, Department of Clinical Sciences Malmö, Lund University, SE 205 02, Jan Waldenströms gata 35, Malmö, Sweden; 3 Population Health Sciences, Bristol Medical School, University of Bristol, Bristol, UK; 4 Medical Research Council Integrative Epidemiology Unit at the University of Bristol, University of Bristol, Bristol, UK; 5 Clinical Studies Sweden, Forum South, Skåne University Hospital, Lund, Sweden; 6 Department of Epidemiology, Harvard T.H. Chan School of Public Health, Boston, MA USA; 7 Department of Public Health and Nursing, NTNU, Norwegian University of Science and Technology, Trondheim, Norway; 8 Department of Endocrinology, St Olav’s Hospital, Trondheim University Hospital, Trondheim, Norway; 9 Department of Clinical Sciences, Umeå University, Umeå, Sweden; 10 Department of Nutrition, Harvard T.H. Chan School of Public Health, Boston, MA USA; 11 NIHR Biomedical Research Centre at the University Hospitals Bristol NHS Foundation Trust, University of Bristol, Bristol, UK; 12 Department of Public Health and Clinical Medicine, Umeå University, Umeå, Sweden

**Keywords:** Risk prediction, Myocardial infarction, Primary prevention, Stroke, Västerbotten Intervention Program

## Abstract

Women with a history of hypertensive disorders of pregnancy (HDP; preeclampsia and gestational hypertension) or delivering low birth weight offspring (LBW; < 2500 g) have twice the risk of cardiovascular disease (CVD). We aimed to study the extent to which history of these pregnancy complications improves CVD risk prediction above and beyond conventional predictors. Parous women attended standardized clinical visits in Sweden. Data were linked to registries of deliveries and CVD. Participants were followed for a first CVD event within 10 years from age 50 (n = 7552) and/or 60 years (n = 5360) and the predictive value of each pregnancy complication above and beyond conventional predictors was investigated. History of LBW offspring was associated with increased risk of CVD when added to conventional predictors in women 50 years of age [Hazard ratio 1.68, 95% Confidence interval (CI) 1.19, 2.37] but not at age 60 (age interaction *p* = 0.04). However, at age 50 years CVD prediction was not further improved by information on LBW offspring, except that a greater proportion of the women who developed CVD were assigned to a higher risk category (categorical net reclassification improvement for events 0.038, 95% CI 0.003, 0.074). History of HDP was not associated with CVD when adjusted for reference model predictors. In conclusion, a history of pregnancy complications can identify women with increased risk of CVD midlife. However, considered with conventional risk factors, history of HDP or having delivered LBW offspring did not meaningfully improve 10-year CVD risk prediction in women age 50 years or older.

**Electronic supplementary material:**

The online version of this article (10.1007/s10654-018-0429-1) contains supplementary material, which is available to authorized users.

## Introduction

Women with a history of hypertensive disorders of pregnancy (HDP; preeclampsia and gestational hypertension) or delivering low birth weight (LBW) offspring have twice the risk of cardiovascular disease (CVD) later in life [1–5]. Current CVD prevention guidelines in the United States [6] and Europe [7] recommend that a woman’s reproductive history should be part of her CVD risk assessment. Complications during pregnancy are associated with earlier development of conventional CVD risk factors [8], increased risk of chronic kidney disease [9] and diabetes mellitus [10], but the strength of the relative risk of pregnancy complications for CVD appears to decline with age [11, 12]. However, it is unknown whether information on prior HDP or LBW offspring improves CVD risk prediction above and beyond current risk prediction models based on conventional CVD risk factors [7, 13, 14].

We investigated the extent to which information on history of HDP or ever delivering LBW offspring added value to the 10-year prediction of CVD in parous middle-aged women. To accomplish this, we predicted the risk of CVD utilizing a conventional prediction model [15] in a population-based cohort with CVD risk factors measured at baseline, and separately evaluated its performance following the inclusion of HDP or LBW offspring history.

## Methods

We used data from a prospectively assessed cohort with standardized clinical assessments in primary care (the Västerbotten Intervention Program) [16, 17] in combination with population-based registries on pregnancy history, in-patient care, and cause of death. Data on emigration were collected from Statistics Sweden (the government agency responsible for the management of Swedish population data). Data were matched via the personal identification number, which is unique for all individuals residing in Sweden [18]. All registry data were collected in accordance with Swedish law and the study was approved by the Ethical Review Board at Lund University, Sweden (2014/337).

### Clinical assessment in primary care

All residents in Västerbotten County (population 264,000, as of June 2016) in Northern Sweden have been, since the early 1990s, invited to visit their primary care provider at ages 50 and 60 years [17]. The goal of these visits is preventive health care with focus on cardiometabolic risk factors. Approximately 50–70% of the eligible population attends. Visits are standardized and generally follow an overnight fast. Further description of clinical visits can be found in the Supplement.

### Pregnancy complication data

For deliveries from 1955 to 1972, we utilized a local birth register covering the county of Västerbotten and the adjacent county Västernorrland. Offspring birth weight was available for all as a binary variable (< 2500 or ≥ 2500 g). For deliveries from 1973 onward, we used the Swedish Medical Birth Register (MBR). Diagnoses during pregnancy were classified according to the International Classification of Disease (ICD): ICD-8 was used until 1986, ICD-9 from 1987 to 1996, and ICD-10 was introduced in 1997. We defined HDP (i.e. preeclampsia/eclampsia/toxemia or gestational hypertension) according to corresponding ICD codes (Supplement). The MBR has been extensively used for research, including investigations on the association between HDP and fetal growth restriction, and maternal CVD [19, 20]. The local birth register and the MBR are further described in the Supplement.

### Cardiovascular disease events

All hospitalizations in Sweden since 1987 are reported to the Swedish National In-Patient Register and diagnoses are registered as ICD codes (ICD-9: 1987–1996, ICD-10: 1996–2014) [21]. Similarly, the Swedish Cause of Death Register captures mortality related diagnoses. For the purpose of this study, we defined incident events (ICD codes given in Supplement) as myocardial infarction, angina, stroke and transient ischemic attack (TIA). When utilizing diagnoses related to deaths, we included the first diagnosis registered and underlying causes of death.

### Study sample

**Fig. 1:**
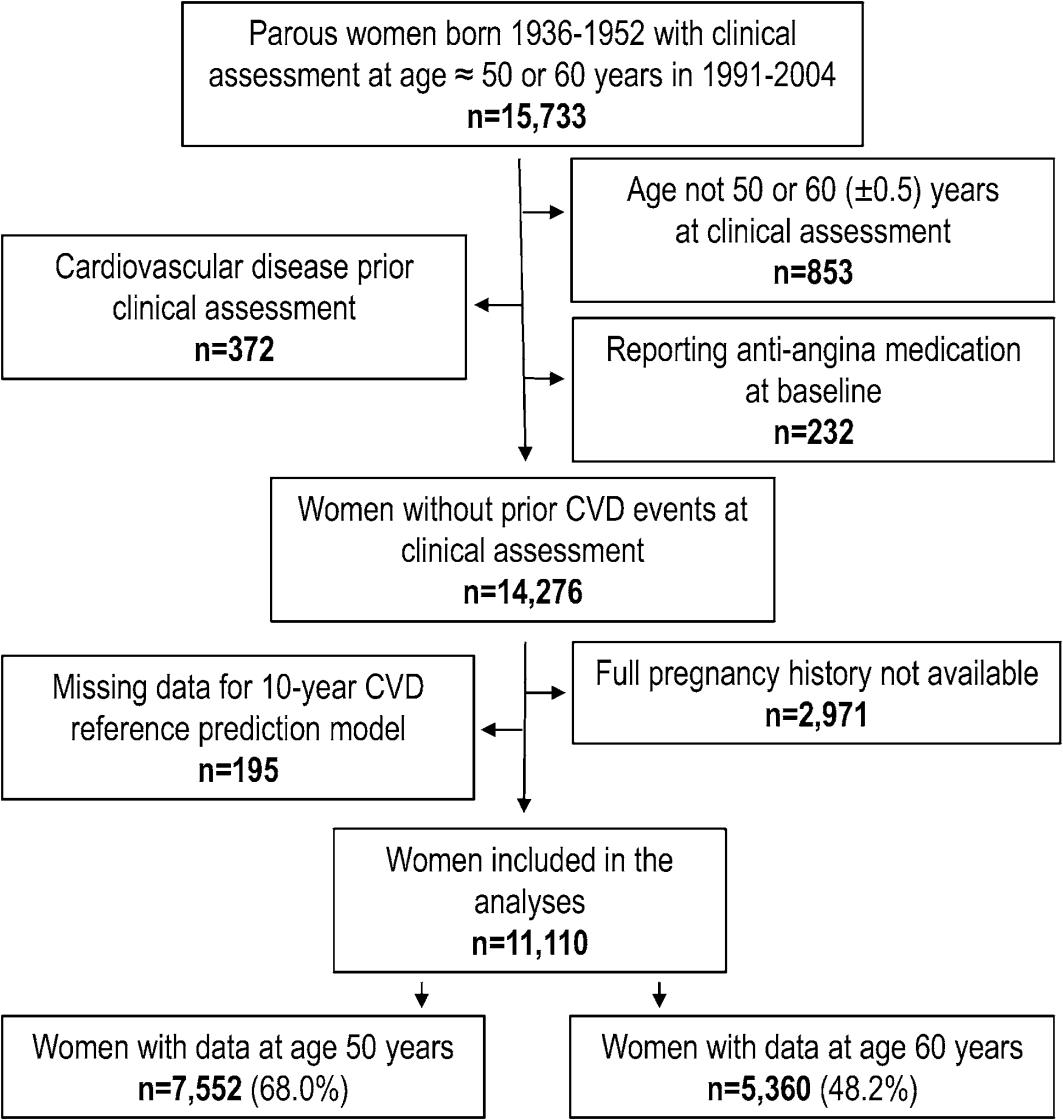
Study sample identification. Flowchart of women included in the study sample. CVD: Cardiovascular disease

Our study sample comprised women born 1936–1952 who visited their primary care provider for a standardized clinical assessment between January 1, 1991 and December 31, 2004, at approximately age 50 or 60 years (Fig. 1). In order to capture full reproductive history, we restricted analyses to those born 1936 and later (i.e. 19 years or younger at the start of pregnancy registration in 1955) and excluded women who did not reside in the geographical area of the local birth registry in young adulthood. Only women with at least one confirmed delivery were included in the analysis. In total, 11,110 parous women (77.8% of those eligible without prior CVD at the clinical assessment) were included in our final study sample. In analyses not stratified by age, these 11,110 women contributed data only from their last clinical assessment that included 10-years of post-assessment follow-up. However, in the analyses stratified by age at clinical assessment, women who had more than one clinical assessment could contribute with data at both ages, resulting in a total of 7552 women observed from age 50 and 5360 women from age 60 years.

### Reference prediction model

We sought to investigate the incremental value of a history of HDP or delivering LBW offspring in CVD risk prediction, building upon established risk factors included in a published prediction model. We chose as the reference model the “lab-based model” published by Gaziano et al. (c-index 0.83), and accordingly replicated that model’s choice, and parameterization, of the conventional risk factors [15]. This model utilized CVD risk factors measured at baseline in our sample [age, log total-cholesterol, log systolic blood pressure, anti-hypertensive medication (yes/no), diabetes mellitus (yes/no), and smoking (yes/no)] and a composite CVD endpoint, including both hard CVD events and diagnoses indicating severe CVD, similar to that defined in this study.

### Statistical analyses

Participants were followed from the clinical assessment until the date of a CVD event, death, emigration, or 10 years from the baseline visit, whichever came first. We used Cox proportional hazards regression models for all prediction analyses. To assess the proportional hazards assumption we used Schoenfeld residual plots and cumulative Martingale residuals. We first studied the association between each pregnancy complication and 10-year CVD in univariate models and then adjusted for all variables included in the reference prediction model. If independently associated with CVD in the latter model, we proceeded to specifically evaluate the predictive value of the pregnancy complication when added to the reference model. As we hypothesized a priori that the pregnancy variables would add more to CVD prediction at younger ages, i.e. at a time point with lower burden of traditional cardiovascular risk factors, we tested multiplicative interactions between age at baseline and each pregnancy complication.

To evaluate model performance, we first estimated the beta coefficients for the reference model by constructing Cox models in the study sample. We refit the reference model also including a term for the relevant pregnancy complication to investigate the incremental value of incorporating this information. Using the observed 10-year survival and estimated betas [22] we calculated the 10-year CVD risk for each participant as predicted by each model.

To describe changes in model discrimination with the addition of the pregnancy complication history, we calculated the difference in c-index and integrated discriminatory improvement (IDI). To report relevant improvement in model risk reclassification, we calculated categorical net reclassification improvement (NRI) separately for cases and non-cases. To reflect current clinical guidelines on primary prevention, we used the following three groups to categorize 10-year CVD risk: < 5, 5–10, and > 10% [23, 24]. Bootstrapping (1000 iterations) was used to estimate 95% confidence intervals (CI) for model comparisons. Tests of calibration (GND test) were performed for all prediction models. Analyses were performed using SAS 9.4 (SAS Institute, Inc., Cary, NC, USA). The publicly-available macros used for prediction model evaluation were developed by Cook et al. [25]. Several additional sub-analyses are described in the Supplement file.

## Results

Table 1 presents baseline characteristics of the study sample by age at clinical assessment as the association between LBW and CVD differed by age (see below). Women assessed at age 60 years had a worse CVD risk profile compared to those assessed at age 50 years, with the exception that smoking was less prevalent at age 60 years. During the 10 years following clinical assessment at age 50 years, 132 (1.7%) women died from non-CVD causes and 15 (0.2%) emigrated; corresponding numbers were 200 (3.7%) and four (0.1%), respectively, for women assessed at age 60 years. Among women age 50 years at baseline, 257 (3.4%) experienced a CVD event within 10 years whereas 405 (7.6%) women followed from age 60 years experienced an event.

**Table 1:** Baseline characteristics of study sample and first CVD event during 10-year follow-up by age at clinical assessment (n = 11110)

Characteristic	Age 50^a^	Age 60^a^
Number of participants	7552 (68.0%)^b^	5360 (48.2%)^b^
Systolic blood pressure, mmHg, median (IQR)	125 (115, 140)	136 (122, 150)
Total serum cholesterol, mmol/L, median (IQR)	5.64 (5.00, 6.38)^c^	5.96 (5.29, 6.70)^d^
Anti-hypertensive medication	811 (10.7)	1214 (22.7)
Current smoker	1851 (24.5)	1007 (18.8)
Diabetes mellitus	66 (0.9)	138 (2.6)
Body mass index, kg/m^2^, median (IQR)	24.7 (22.7, 27.6)	25.9 (23.5, 28.9)
Parity		
1	2926 (38.7)	1678 (31.3)
2	3394 (44.9)	2646 (49.4%)
3	981 (13.0)	833 (15.5)
≥ 4	251 (3.3)	203 (3.8)
Ever hypertensive disorder of pregnancy	328 (4.3)	285 (5.3)
Ever low birth weight offspring (< 2500 g)	624 (8.3)	373 (7.0)
First CVD event during 10 years of follow-up	257 (3.4)	405 (7.6)
Myocardial infarction	101 (1.3)	149 (2.8)
Angina	67 (0.9)	93 (1.7)
Stroke	72 (1.0)	125 (2.3)
TIA	22 (0.3)	46 (0.9)

Presented as n (%) unless otherwise noted

*CVD* cardiovascular disease, *IQR* interquartile range, *TIA* transient ischemic attack

^a^ ± 0.5 years

^b^A subset of participants attended clinical visits at both age 50 and 60 years

^c^n = 5666 participants (75.0%) with hypercholesterolemia (≥ 5.0 mmol/l) and 37 (0.5%) with lipid lowering medication at age 50 years

^d^n = 4488 participants (83.7%) with hypercholesterolemia (≥ 5.0 mmol/l) and 167 (3%) with lipid lowering medication at age 60 years

### Association between predictors at clinical assessment and 10-year CVD incidence

In the univariate analysis, both history of HDP (HR = 1.90, 95% CI 1.20, 2.99) and LBW offspring (HR = 1.95, 95% CI 1.38, 2.75) were associated with 10-year CVD in women age 50. However, there were no similar associations in women age 60 years for HDP (HR = 1.04, 95% CI 0.68, 1.60, *p* = 0.05 for interaction by age) or LBW offspring (HR = 1.04, 95% CI 0.71, 1.51, *p* = 0.03 for interaction by age). When adjusted for reference model predictors, LBW was associated with CVD at age 50 years (Table 2, HR = 1.68, 95% CI 1.19, 2.37) but not at age 60 years (Table 3, HR = 0.94, 95% CI 0.65, 1.38, *p* = 0.04 for interaction by age). When adjusted for reference model predictors, history of HDP was not associated with 10-year CVD in either the age stratified models (Table 2 and Table S1) or the non-age stratified model (Table S2). History of HDP was therefore not examined further in the main analysis.

**Table 2:** Estimates for 10-year CVD model predictors among women assessed at age 50 (n = 7552) by added pregnancy complication

Reference model + Low birth weight offspring (< 2500 g)				
Predictor	Beta	SE	Hazard ratio (95% CI)	*p* value
Log total cholesterol	1.19	0.33	3.28 (1.72, 6.24)	< 0.001
Log systolic blood pressure	3.22	0.45	25.1 (10.5, 60.2)	< 0.001
Anti-hypertensive medication	0.51	0.16	1.66 (1.22, 2.26)	0.001
Current smoker	0.83	0.13	2.30 (1.79, 2.96)	< 0.001
Diabetes mellitus	0.99	0.36	2.69 (1.32, 5.51)	0.007
Low birth weight offspring	0.52	0.18	1.68 (1.19, 2.37)	0.003

Reference model + Hypertensive disorders of pregnancy				
Predictor	Beta	SE	Hazard ratio (95% CI)	*p* value
Log total cholesterol	1.19	0.33	3.29 (1.72, 6.27)	< 0.001
Log systolic blood pressure	3.20	0.45	24.4 (10.1, 59.1)	< 0.001
Anti-hypertensive medication	0.51	0.16	1.67 (1.22, 2.29)	0.001
Current smoker	0.85	0.13	2.34 (1.82, 3.01)	< 0.001
Diabetes mellitus	1.02	0.37	2.77 (1.35, 5.67)	0.005
Hypertensive disorders of pregnancy	0.17	0.24	1.19 (0.74, 1.90)	0.48

*CI* Confidence interval

**Table 3:** Risk reclassification for 10-year CVD prediction in women age 50 years with low birth weight offspring added to the reference model

Women with CVD events during 10-year follow-up				
Reference model + LBW offspring (< 2500 g)				
Reference model	0 to < 5%	5 to < 10%	≥ 10%	Total
0 to < 5%	138 (92.6)	11 (7.4)	0	149 (58.0)
5 to < 10%	5 (7.5)	57 (85.1)	5 (7.5)	67 (26.1)
≥ 10%	0	1 (2.4)	40 (97.6)	41 (16.0)
Total	143 (55.6)	69 (26.9)	45 (17.5)	257

Women with no CVD events during 10-year follow-up				
Reference model + LBW offspring (< 2500 g)				
Reference model	0 to < 5%	5 to < 10%	≥ 10%	Total
0 to < 5%	5828 (98.0)	119 (2.0)	0	5947 (83.2)
5 to < 10%	128 (12.9)	806 (81.3)	58 (5.9)	992 (13.9)
≥ 10%	0	38 (18.2)	171 (81.8)	209 (2.9)
Total	5956 (83.3)	963 (13.5)	229 (3.2)	7148

Data presented as n (%). Women censored due to non-events within 10-years of baseline are excluded from the table (n = 147). Categorical NRI for events = 0.038 (95% CI 0.003, 0.074, *p* = 0.04). Categorical NRI for non-events = − 0.001 (95% CI − 0.006, 0.003, *p* = 0.63). IDI = 0.0014 (95% CI − 0.0002, 0.0032, *p* = 0.10). C-index reference model = 0.69 (95% CI 0.66, 0.72). C-index reference model + LBW = 0.70 (95% CI 0.66, 0.73). C-index difference = 0.01 (95% CI − 0.0003, 0.02)

*CI* Confidence interval, *CVD* cardiovascular disease, *IDI* integrated discriminatory improvement, *LBW* low birth weight (< 2500 g), *NRI* net reclassification improvement

### Incremental value of LBW offspring in CVD prediction when added to the reference model

Table 3 presents the risk reclassification separately for events and non-events, as well as discrimination statistics for adding LBW offspring to the reference model in women age 50 years. A greater proportion of the women who developed CVD were assigned to a higher risk category (categorical NRI for events 0.038; 95% CI 0.003, 0.074) but the risk classification among women without an event remained constant (categorical NRI for non-events: − 0.001; 95% CI − 0.006, 0.003). The reference model had adequate discriminatory performance (c-index: 0.69, 95% CI 0.66, 0.72) but adding information on LBW did not improve discrimination further. All prediction models were adequately calibrated as tested by the GND test (*p* > 0.05).

### Additional results

Supplementary results, including risk reclassification tables for HDP and for overall analyses (i.e. not stratified by age) can be found in the Supplement (Table S3 to Table S8). These analyses supported the results of the main analysis presented here. In addition, the sensitivity analyses described in the Supplement, including restricting the outcome to hard CVD events, investigating 7.5% 10-year CVD risk as cut-off, or restricting HDP diagnoses to diagnoses related to preeclampsia, supported the main results.

## Discussion

In this study of parous women, both history of HDP and having delivered LBW offspring were associated with increased risk of CVD at age 50 but not 60 years. However, when added to conventional CVD risk factors, 10-year CVD risk prediction was not meaningfully improved. To our knowledge, this is the first comprehensive investigation of the incremental value of history of HDP or LBW offspring for predicting 10-year risk of CVD. Parikh et al. [26] tested the clinical value of adding a range of reproductive factors, but not HDP or LBW, to a prediction model that included conventional predictors in a sample of mostly older middle-aged women. Discrimination and risk reclassification for non-cases, but not for cases, were slightly improved.

The crude association between each pregnancy complication and 10-year CVD at age 50 years is consistent with the two-fold increased risk reported in previous studies [1–5]. The observed null association between HDP and CVD in women 60 years of age in this cohort could potentially be due to less adequate diagnoses at the time of pregnancy, as these women partly belong to a different birth cohort, compared to women with a clinical examination at age 50 years. However, a similar pattern is evident also for LBW offspring, which, being defined only by offspring birth weight, might be less sensitive to temporal changes in definition than a clinical diagnosis of HDP.

Though the c-statistics for the reference model might appear modest, it is important to consider that age is a strong predictor of CVD. Many previous studies of CVD prediction have reported c-statistics close to 0.8, but include a wider and continuous age range of participants than this sample [15, 22, 27–29] However, with age being a strong risk factor for CVD, an age restricted sample results in lower discrimination. A relevant comparison is a study including men approximately age 71 years by Zethelius et al. [30] which reported a C-statistic of 0.69 for 10-year risk of CVD death in a model with conventional CVD predictors.

### Strengths and limitations

This study has several strengths that should be noted: a previously published CVD prediction model was utilized as reference, few women were censored during the 10-year intervals, and the baseline data collection was standardized. Though our sample is not a naïve treatment cohort without preventive measures during follow-up, it reflects primary health care settings in which conventional CVD risk factors are collected and treated if appropriate. More importantly, only recently has the topic of pregnancy complications been featured in European CVD prevention guidelines, appearing in the European Society of Cardiology guidelines for CVD prevention for the first time in 2016 [7]. Thus, the treatment of the study sample throughout follow-up is likely to be unbiased in this regard as history of HDP or LBW offspring were unlikely to directly affect any preventive health care received. However, there are also some limitations. We did not have data on high-density lipoprotein (HDL) or C-reactive protein (CRP) on all participants, which prevented us from utilizing several CVD prediction models as Refs. [27–29, 31] Nevertheless, if history of HDP and LBW offspring do not provide incremental value to our prediction model without HDL, this information is not likely to further improve prediction models with even better performance. All women in the sample were age 50–60 years, limiting generalizability to younger women, and we did not have data on current menopausal status. However, menopause is generally not considered in clinical CVD prediction models in women. Strictly speaking, the generalizability is also limited to women who were living in the geographical area of the pregnancy registry from young adulthood and attended the clinical visits in middle age. However, it seems unlikely that the exclusion of women with incomplete reproductive history based on relocation (e.g. pursuing higher education) would influence our results. While many studies on pregnancy complications and later CVD have relied on registry-based data [2, 3, 19], chart review confirmation is more common in studies of CVD prediction. Here, the pregnancy exposures and CVD outcomes were ascertained via registry based ICD code data and not through chart review. For the purpose of this study, the validation of in-patient registry based diagnoses of stroke and myocardial infarction appears to be adequate (positive predictive value generally > 80%) [21]. Furthermore, the registry-based prospective follow-up for events results in < 4% of participants lost to follow-up.

### Implications for future research

Whereas we did not find meaningful utility of incorporating history of HDP or LBW offspring in CVD prediction in this population of middle-aged parous women of Scandinavian ethnicity, this topic warrants evaluation in other populations that also include high-quality data on gestational age and gestational diabetes mellitus, both of which have been associated with maternal CVD [1]. In particular, preterm delivery appears to be associated with CVD independently of the cardiovascular risk factors included in CVD risk prediction models [32], and could improve CVD prediction better than HDP or LBW. As the clinical diagnoses of HDP are likely to have improved since the 1950–1960s, e.g. due to the clinical implementation of urine dipsticks to detect proteinuria and through consensus guidelines, it cannot be excluded that HDP diagnoses set according to these more strict and defined definitions might have greater relevance for CVD prediction in women of younger generations.

This study did not include nulliparous women. Whereas one study found that nulliparity was associated with increased risk of CVD in women [33], another study did not find an association between ever being pregnant and coronary heart disease when adjusting for established CVD risk factors [26]. Nonetheless, to study the clinical utility of pregnancy complications in predicting CVD in all women, future studies should preferably include women regardless of parity. Also, the incremental value of pregnancy complications in predicting CVD might differ by race/ethnicity given the difference in prevalence of pregnancy complications [34], and the reported interaction between ethnicity/race, pregnancy complications, and age on risk of CVD [14].

Finally, we note that pregnancy complication history might be more predictive of 10-year CVD risk before age 50, at which point CVD risk factors such as hypertension have emerged. Similarly, pregnancy complications at age 20, 30 or 40 years may predict longer-term CVD risk over several decades. While the current study suggests that history of HDP or LBW offspring does little to predict CVD risk in addition to already established CVD risk factors at age 50 or 60 years, history of pregnancy complications may still be useful to identify young women to prevent the development of the hypertension, dyslipidemia, overweight and diabetes with which these pregnancy complications are associated.

## Conclusion

In this cohort of women aged 50 and 60 years, information on prior HDP or LBW offspring did not meaningfully improve 10-year CVD risk prediction when added to an established prediction model that already accounted for conventional CVD risk measured at those ages. This suggests that, for women age 50 and older, pregnancy complication history does not add to CVD risk stratification. However, at younger ages not tested with this study, pregnancy complication history is known to predict the development of conventional CVD risk factors [1, 13, 14], and may still improve clinical risk prediction before age 50. Therefore, these factors should still be evaluated in CVD risk prediction models in younger women, as well as in other populations.

## Electronic supplementary material

Below is the link to the electronic supplementary material.

Supplementary material 1 (DOCX 55 kb)

## References

[1] Rich-Edwards JW, Fraser A, Lawlor DA, Catov JM (2014). Pregnancy characteristics and women’s future cardiovascular health: an underused opportunity to improve women’s health?. Epidemiol Rev.

[2] Ray JG, Vermeulen MJ, Schull MJ, Redelmeier DA (2005). Cardiovascular health after maternal placental syndromes (CHAMPS): population-based retrospective cohort study. The Lancet..

[3] Smith GC, Pell JP, Walsh D (2001). Pregnancy complications and maternal risk of ischaemic heart disease: a retrospective cohort study of 129,290 births. The Lancet.

[4] Friedlander Y, Paltiel O, Manor O, Deutsch L, Yanetz R, Calderon R (2007). Birthweight of offspring and mortality of parents: the Jerusalem perinatal study cohort. Ann Epidemiol.

[5] Brown MC, Best KE, Pearce MS, Waugh J, Robson SC, Bell R (2013). Cardiovascular disease risk in women with pre-eclampsia: systematic review and meta-analysis. Eur J Epidemiol.

[6] Mosca L, Benjamin EJ, Berra K, Bezanson JL, Dolor RJ, Lloyd-Jones DM (2011). Effectiveness-based guidelines for the prevention of cardiovascular disease in women—2011 update a guideline from the American heart association. J Am Coll Cardiol.

[7] Piepoli MF, Hoes AW, Agewall S, Albus C, Brotons C, Catapano AL (2016). 2016 European Guidelines on cardiovascular disease prevention in clinical practiceThe Sixth Joint Task Force of the European Society of Cardiology and Other Societies on Cardiovascular Disease Prevention in Clinical Practice (constituted by representatives of 10 societies and by invited experts)Developed with the special contribution of the European Association for Cardiovascular Prevention & Rehabilitation (EACPR). Eur Heart J.

[8] Paauw ND, Luijken K, Franx A, Verhaar MC, Lely AT (2016). Long-term renal and cardiovascular risk after preeclampsia: towards screening and prevention. Clin Sci.

[9] Vikse BE, Irgens LM, Leivestad T, Skjærven R, Iversen BM (2008). Preeclampsia and the risk of end-stage renal disease. N Engl J Med.

[10] Feig DS, Shah BR, Lipscombe LL, Wu CF, Ray JG, Lowe J (2013). Preeclampsia as a risk factor for diabetes: a population-based cohort study. PLoS Med.

[11] Cirillo PM, Cohn BA (2015). Pregnancy complications and cardiovascular disease death 50-year follow-up of the child health and development studies pregnancy cohort. Circulation.

[12] Nelander M, Cnattingius S, Åkerud H, Wikström J, Pedersen NL, Wikström A-K (2016). Pregnancy hypertensive disease and risk of dementia and cardiovascular disease in women aged 65 years or older: a cohort study. BMJ Open.

[13] Seely EW, Tsigas E, Rich-Edwards JW (2015). Preeclampsia and future cardiovascular disease in women: how good are the data and how can we manage our patients?. Semin Perinatol.

[14] Ahmed R, Dunford J, Mehran R, Robson S, Kunadian V (2014). Pre-eclampsia and future cardiovascular risk among women: a review. J Am Coll Cardiol.

[15] Gaziano TA, Young CR, Fitzmaurice G, Atwood S, Gaziano JM (2008). Laboratory-based versus non-laboratory-based method for assessment of cardiovascular disease risk: the NHANES I follow-up study cohort. Lancet.

[16] Hallmans G, Agren A, Johansson G, Johansson A, Stegmayr B, Jansson J-H (2003). Cardiovascular disease and diabetes in the Northern Sweden Health and Disease Study Cohort—evaluation of risk factors and their interactions. Scand J Public Health Suppl.

[17] Norberg M, Wall S, Boman K, Weinehall L (2010). The Vasterbotten Intervention Programme: background, design and implications. Glob Health Action.

[18] Ludvigsson JF, Otterblad-Olausson P, Pettersson BU, Ekbom A (2009). The Swedish personal identity number: possibilities and pitfalls in healthcare and medical research. Eur J Epidemiol.

[19] Wikström A-K, Haglund B, Olovsson M, Lindeberg SN (2005). The risk of maternal ischaemic heart disease after gestational hypertensive disease. BJOG Int J Obstet Gynaecol.

[20] Bonamy A-KE, Parikh NI, Cnattingius S, Ludvigsson JF, Ingelsson E (2011). Birth characteristics and subsequent risks of maternal cardiovascular diseaseclinical perspective. Circulation.

[21] Ludvigsson JF, Andersson E, Ekbom A, Feychting M, Kim J-L, Reuterwall C (2011). External review and validation of the Swedish national inpatient register. BMC Public Health.

[22] D’Agostino RB, Vasan RS, Pencina MJ, Wolf PA, Cobain M, Massaro JM (2008). General cardiovascular risk profile for use in primary care the framingham heart study. Circulation.

[23] Stone NJ, Robinson JG, Lichtenstein AH, Bairey Merz CN, Blum CB, Eckel RH (2014). 2013 ACC/AHA Guideline on the Treatment of Blood Cholesterol to Reduce Atherosclerotic Cardiovascular Risk in Adults: A Report of the American College of Cardiology/American Heart Association Task Force on Practice Guidelines. J Am Coll Cardiol.

[24] Cardiovascular disease: risk assessment and reduction, including lipid modification | 1-recommendations | Guidance and guidelines | NICE [Internet]. [cited 2016 Sep 16]. https://www.nice.org.uk/guidance/cg181/chapter/1-recommendations?unlid=694096047201695142932.

[25] Cook N. SAS Macros [Internet]. [cited 2016 Dec 15]. http://ncook.bwh.harvard.edu/sas-macros.html.

[26] Parikh NI, Jeppson RP, Berger JS, Eaton CB, Kroenke CH, LeBlanc ES (2016). Reproductive risk factors and coronary heart disease in the women’s health initiative observational study. Circulation.

[27] Ridker PM, Buring JE, Rifai N, Cook NR (2007). Development and validation of improved algorithms for the assessment of global cardiovascular risk in women: the Reynolds risk score. JAMA.

[28] Goff J David C, Lloyd-Jones DM, Bennett G, Coady S, D’Agostino S Ralph B, Gibbons R, et al. 2013 ACC/AHA Guideline on the assessment of cardiovascular risk a report of the American College of Cardiology/American Heart Association Task Force on Practice Guidelines. J Am Coll Cardiol [Internet]. 2014 [cited 2016 Nov 23];63. 10.1016/j.jacc.2013.11.005. PMC4700825 24239921

[29] Hippisley-Cox J, Coupland C, Vinogradova Y, Robson J, Minhas R, Sheikh A (2008). Predicting cardiovascular risk in England and Wales: prospective derivation and validation of QRISK2. BMJ.

[30] Zethelius B, Berglund L, Sundström J, Ingelsson E, Basu S, Larsson A (2008). Use of multiple biomarkers to improve the prediction of death from cardiovascular causes. N Engl J Med.

[31] Conroy RM, Pyörälä K, Fitzgerald AP, Sans S, Menotti A, Backer GD (2003). Estimation of ten-year risk of fatal cardiovascular disease in Europe: the SCORE project. Eur Heart J.

[32] Tanz LJ, Stuart JJ, Williams PL, Rimm EB, Missmer SA, Rexrode KM (2017). Preterm delivery and maternal cardiovascular disease in young and middle-aged adult women clinical perspective. Circulation.

[33] Parikh NI, Cnattingius S, Dickman PW, Mittleman MA, Ludvigsson JF, Ingelsson E (2010). Parity and risk of later-life maternal cardiovascular disease. Am Heart J.

[34] Tanaka M, Jaamaa G, Kaiser M, Hills E, Soim A, Zhu M (2007). Racial disparity in hypertensive disorders of pregnancy in New York state: a 10-year longitudinal population-based study. Am J Public Health.

