# Supplementary material for: The value of pregnancy complication history for 10-year cardiovascular disease risk prediction in middle-aged women
Source: Eur J Epidemiol. 2018 Jul 30;33(10):1003–10. doi: 10.1007/s10654-018-0429-1 (PMC6153555; doi:10.1007/s10654-018-0429-1)
Supplement: Supplementary file 1 — Supplementary material 1 (DOCX 55 kb) [file 10654_2018_429_MOESM1_ESM.docx]

**SUPPLEMENTAL MATERIAL**

**European Journal of Epidemiology**

**The value of pregnancy complication history for 10-year cardiovascular disease risk prediction in middle-aged women**

Timpka et al.

**Corresponding author:**

Simon Timpka, Lund University, Malmö, Sweden

[simon.timpka@med.lu.se](mailto:simon.timpka@med.lu.se)

**Additional information about clinical assessment in primary care**

A nurse administers a questionnaire and performs clinical measurements. Current medications (including anti-hypertensives, anti-angina, and anti-diabetic medications), smoking (yes/no), and previously diagnosed diabetes mellitus are reported via a self-administered questionnaire. During the time period in which data for this study were collected, systolic blood pressure was measured with a mercury sphygmomanometer with the patient in a supine position following a five minute rest. Reflotron bench-top analyzers (Roche Diagnostics) were used for all analyses of total serum cholesterol and were calibrated in accordance with guidelines from the External Quality Assurance in Laboratory Medicine in Sweden – EQUALIS.

**Additional information about local birth register data 1955 to 1972**

We utilized a birth register that includes pregnancies/deliveries in the two counties Västernorrland and Västerbotten during the years 1955 to 1972 in northern Sweden. [1] In summary, Umeå University, Sweden, and the Swedish Board of Health and Welfare undertook an effort in 1984 to retrospectively collect information from all maternal wards in the area (in Västerbotten; n=16 in 1955). All ledgers but two from minor units in Västerbotten were obtained; the first had been lost when the building was destroyed in a fire and the second had been discarded as only seven deliveries had occurred at this particular unit. All maternal and offspring diagnoses were coded according to ICD-8. The registry information has previously been used for several epidemiological publications on preeclampsia and low birth weight. [2] [3] [4] At some point since the late 1990s the local delivery register data was lost at the Board of Health and Welfare. However, for the purpose of this study we have worked with Statistics Sweden (a government agency holding large population-based databases) to match women and offspring to unique personal identification numbers [5] utilizing a de-identified secondary version of the data. Through the use of registry data available in this de-identified file, such as date of birth and maternal parish of residence at delivery, we were able to match the mother to her unique personal identification number for 99.2% of all deliveries. The matching process was performed at Statistics Sweden and the research group only had access to de-identified data.

**Additional information about the Swedish Medical Birth Register 1973-**

The positive predictive value of a HDP diagnosis appears to have increased over time in Sweden, i.e. the quality of the diagnosis recorded in the register has improved. Validation of diagnoses of HDP from the Medical Birth Register from the 1990s [6] showed high positive predictive value for gestational hypertension (84%) and preeclampsia (93%). However, in one study that separated validated records of preeclampsia by year of pregnancy the positive predictive value varied from 68% in 1973-1986 to 93% in 1987-1993. [7] Other validation studies in similar birth registry data from Norway, dating as far back as 1967, also report higher positive predictive value for preeclampsia over time. [8] However, there was indication of substantial overlap between preeclampsia and gestational hypertension and we therefore in this study investigated any HDP as one of two main exposures in our main analyses.

**International classification of disease codes used in the analysis**

*Hypertensive disorders of pregnancy*

ICD-8: 637.01, 637.03, 637.04, 637.09, 637.10, 661.2; ICD-9: 642A, 642D, 642E, 642F, 642G, 642X; ICD-10: O13, O14, O15

*Cardiovascular disease events*

Myocardial infarction: ICD-9: 410; ICD-10: I21

Angina: ICD-9: 411, 413; ICD-10: I20

Stroke: ICD-9: 431, 434, 436; ICD-10: I61, I63, I64

Transient ischemic attack (TIA): ICD-9: 435; ICD-10: G45.9

**Additional analyses**

A potentially important clinical threshold for CVD risk is 7.5% 10-year risk of CVD (when statin therapy is recommended by the American College of Cardiology and American Heart Association). [9] Therefore, we in addition investigated the impact of using this cut-off on the categorical NRI. Furthermore, we investigated the incremental value of history of HDP or LBW offspring when restricting the outcome to myocardial infarction and stroke. We also tested whether restricting the HDP exposure to diagnoses related to preeclampsia/toxemia/eclampsia could enhance prediction or if our results changed when we accounted for current lipid-lowering medication.

**Supplemental Results**

Supplementary results from the main analysis are shown in Table S1 to Table S8. These results supported those shown in the main manuscript. In addition, there was no clinically relevant risk reclassification when we investigated the threshold of 7.5% 10-year CVD risk and restricting the outcome to myocardial infarction and stroke events did not substantially alter results. Likewise, restricting exposure to preeclampsia-related diagnoses or including current lipid lowering medication in the prediction model did not alter results (data not shown).

Of 5,360 women who attended the 60 years clinical visit, 2,555 women were born 1941-1944 and had the opportunity to attend a clinical visit at age 50 years as well. Characteristics of women who attended clinic at age 60 years did not differ between these women who did (64.3%) and did not attend the 50 year clinic (35.7%), except for the prevalence of smokers which was lower amongst those who did attend the 50 year clinic (17.0% vs. 23.7%).

**Supplemental tables**

**Table S1.** Estimates for 10-year CVD model predictors among women assessed at age 60 (n=5,360) by added pregnancy complication

**Table S2**. The multivariate association between pregnancy complications and cardiovascular disease during 10-years of follow-up when last available clinical assessment was used for each woman (n=11,110)

**Table S3.** The multivariate Cox regression estimates for predictors in the reference prediction model stratified by age at baseline

**Table S4.** Risk reclassification for 10-year CVD prediction in women age 60 years with low birth weight offspring added to the reference model

**Table S5.** Risk reclassification for 10-year CVD prediction in women age 50 years with history of hypertensive disorders of pregnancy added to the reference model

**Table S6.** Risk reclassification for 10-year CVD prediction in women age 60 years with history of hypertensive disorders of pregnancy added to the reference model

**Table S7.** Risk reclassification for 10-year CVD prediction with low birth weight offspring added to the reference model not stratified by age at baseline

**Table S8.** Risk reclassification for 10-year CVD prediction with history of hypertensive disorders of pregnancy added to the reference model not stratified by age at baseline

| **Table S1.** Estimates for 10-year CVD model predictors among women assessed at age 60 (n=5,360) by added pregnancy complication | | | | |
| --- | --- | --- | --- | --- |
| **Reference model + Low birth weight offspring (<2,500 g)** | | | | |
| **Predictor** | **Beta** | **Standard error** | **Hazard ratio (95% CI)** | **p-value** |
| Log total cholesterol | 0.37 | 0.25 | 1.45 (0.89, 2.34) | 0.13 |
| Log systolic blood pressure | 1.93 | 0.37 | 6.87 (3.33, 14.2) | <0.001 |
| Anti-hypertensive medication | 0.40 | 0.11 | 1.49 (1.20, 1.85) | <0.001 |
| Current smoker | 0.65 | 0.11 | 1.92 (1.55, 2.39) | <0.001 |
| Diabetes mellitus | 0.69 | 0.22 | 1.99 (1.30, 3.06) | 0.001 |
| LBW offspring | -0.06 | 0.19 | 0.94 (0.65, 1.38) | 0.75 |
| **Reference model + Hypertensive disorders of pregnancy** | | | | |
| **Predictor** | **Beta** | **Standard error** | **Hazard ratio (95% CI)** | **p-value** |
| Log total cholesterol | 0.37 | 0.25 | 1.45 (0.89, 2.34) | 0.13 |
| Log systolic blood pressure | 1.94 | 0.37 | 6.98 (3.38, 14.4) | <0.001 |
| Anti-hypertensive medication | 0.41 | 0.11 | 1.50 (1.21, 1.87) | <0.001 |
| Current smoker | 0.65 | 0.11 | 1.91 (1.54, 2.38) | <0.001 |
| Diabetes mellitus | 0.71 | 0.22 | 2.03 (1.32, 3.13) | 0.001 |
| HDP | -0.19 | 0.22 | 0.83 (0.53, 1.28) | 0.39 |
| CI: Confidence interval; HDP: Hypertensive disorders of pregnancy; LBW: Low birth weight (<2,500 g) | | | | |

| **Table S2.** The multivariate association between pregnancy complications and CVD during 10-years of follow-up when last available clinical assessment was used for each woman (n=11,110) | | | | |
| --- | --- | --- | --- | --- |
| **Pregnancy complication** | **Main effect,**  **HR (95% CI)** | **Age,**  **HR (95% CI)** | **Interaction term age,**  **HR (95% CI)** | **p interaction** |
| HDP | 1.37 (0.82, 2.30) | 1.72 (1.43, 2.06) | 0.58 (0.30, 1.14) | 0.11 |
| LBW offspring | 1.63 (1.11, 2.39) | 1.77 (1.47, 2.13) | 0.57 (0.34, 0.98) | 0.04 |
| CVD: Cardiovascular disease; HDP: Hypertensive disorders of pregnancy; HR: Hazard ratio; LBW: Low birth weight (<2,500 g)  events=616; censored within ten years=349  Cox proportional hazards models including terms for the pregnancy complication, age at baseline (50 or 60 years), and their interaction, as well as systolic blood pressure, anti-hypertensive medication, serum-cholesterol, diabetes mellitus, and smoking | | | | |

| **Table S3.** The multivariate Cox regression estimates for predictors in the reference prediction model stratified by age at baseline | | | | |
| --- | --- | --- | --- | --- |
| **Age 50 years** | | | | |
| **Predictor** | **Beta** | **Standard error** | **Hazard ratio (95% CI)** | **p-value** |
| Log total cholesterol | 1.20 | 0.33 | 3.31 (1.73, 6.31) | <0.001 |
| Log systolic blood pressure | 3.23 | 0.45 | 25.4 (10.6, 60.9) | <0.001 |
| Anti-hypertensive medication | 0.53 | 0.16 | 1.70 (1.25, 2.32) | <0.001 |
| Current smoker | 0.85 | 0.13 | 2.34 (1.83, 3.01) | <0.001 |
| Diabetes mellitus | 1.04 | 0.36 | 2.83 (1.38, 5.77) | 0.004 |
| **Age 60 years** | | | | |
| **Predictor** | **Beta** | **Standard error** | **Hazard ratio (95% CI)** | **p-value** |
| Log total cholesterol | 0.37 | 0.25 | 1.45 (0.89, 2.35) | 0.13 |
| Log systolic blood pressure | 1.92 | 0.37 | 6.85 (3.32, 14.1) | <0.001 |
| Anti-hypertensive medication | 0.40 | 0.11 | 1.49 (1.20, 1.85) | <0.001 |
| Current smoker | 0.65 | 0.11 | 1.92 (1.54, 2.39) | <0.001 |
| Diabetes mellitus | 0.69 | 0.22 | 1.99 (1.30, 3.05) | 0.002 |
| CI: Confidence interval | | | | |

| **Table S4.** Risk reclassification for 10-year CVD prediction in women age 60 years with low birth weight offspring added to the reference model | | | | | | |
| --- | --- | --- | --- | --- | --- | --- |
| **Women with CVD events during 10-year follow-up** | | | | | | |
|  | **Reference model + LBW offspring (<2,500 g)** | | | | | |
| **Reference model** | **0 to <5%** | | **5 to <10%** | | **≥10%** | **Total** |
| **0 to <5%** | 53 (98.2) | | 1 (1.9) | | 0 | 54 (13.3) |
| **5 to <10%** | 0 | | 204 (98.6) | | 3 (1.5) | 207 (51.1) |
| **≥10%** | 0 | | 2 (1.4) | | 142 (98.6) | 144 (35.6) |
| **Total** | 53 (13.1) | | 207 (51.1) | | 145 (35.8) | 405 |
| **Women with no CVD events during 10-year follow-up** | | | | | | |
|  | | **Reference model + LBW offspring (<2,500 g)** | | | | |
| **Reference model** | | **0 to <5%** | | **5 to <10%** | **≥10%** | **Total** |
| **0 to <5%** | 1,157 (98.9) | | 13 (1.1) | | 0 | 1,170 (24.6) |
| **5 to <10%** | 14 (0.5) | | 2,694 (99.2) | | 9 (0.3) | 2,717 (57.2) |
| **≥10%** | 0 | | 10 (1.2) | | 854 (98.8) | 864 (18.2) |
| **Total** | 1,171 (24.7) | | 2,717 (57.2) | | 863 (18.2) | 4,751 |
| CI: Confidence interval; CVD: Cardiovascular disease; IDI: Integrated discriminatory improvement; LBW: Low birth weight (<2,500 g); NRI: Net reclassification improvement  Data presented as n (percentage)  Women censored due to non-events within ten years of baseline are excluded from the table (n=204)  Categorical NRI for events = 0.0049 (95% CI: -0.01, 0.02, p = 0.38) Categorical NRI for non-events = 0.0002 (95% CI: -0.003, 0.003, p = 0.88)  IDI = 0.00 (95% CI: -0.0001, 0.0001, p = 0.99) C-index reference model = 0.63 (95% CI: 0.61, 0.66)  C-index reference model + LBW offspring = 0.63 (95% CI: 0.61, 0.66) C-index difference = 0.0004 (95% CI: -0.0005, 0.0012) | | | | | | |

| **Table S5.** Risk reclassification for 10-year CVD prediction in women age 50 years with history of hypertensive disorders of pregnancy added to the reference model | | | | | | | |
| --- | --- | --- | --- | --- | --- | --- | --- |
| **Women with CVD events during 10-year follow-up** | | | | | | | |
|  | **Reference model + history of HDP** | | | | | |  |
| **Reference model** | **0 to <5%** | | **5 to <10%** | | **≥10%** | | **Total** |
| **0 to <5%** | 146 (98.0) | | 3 (2.0) | | 0 | | 149 (58.0) |
| **5 to <10%** | 1 (1.5) | | 65 (97.0) | | 1 (1.5) | | 67 (26.1) |
| **≥10%** | 0 | | 0 | | 41 (100.0) | | 41 (16.0) |
| **Total** | 147 (57.2) | | 68 (26.5) | | 42 (16.3) | | 257 |
| **Women with no CVD events during 10-year follow-up** | | | | | | | |
|  | | **Reference model + history of HDP** | | | | | |
| **Reference model** | | **0 to <5%** | | **5 to <10%** | | **≥10%** | **Total** |
| **0 to <5%** | 5,922 (99.6) | | 25 (0.4) | | 0 | | 5,947 (83.2) |
| **5 to <10%** | 16 (1.6) | | 964 (97.2) | | 12 (1.2) | | 992 (13.9) |
| **≥10%** | 0 | | 10 (4.8) | | 199 (95.2) | | 209 (2.9) |
| **Total** | 5,938 (83.1) | | 999 (14.0) | | 211 (3.0) | | 7,148 |
| CI: Confidence interval; CVD: Cardiovascular disease; HDP: Hypertensive disorders of pregnancy; IDI: Integrated discriminatory improvement; NRI: Net reclassification improvement  Data presented as n (percentage)  Women censored due to non-events within ten years of baseline are excluded from the table (n=147)  Categorical NRI for events = 0.01 (95% CI: -0.004, 0.03, p = 0.18) Categorical NRI for non-events = -0.002 (95% CI: -0.003, 0.001, p = 0.14)  IDI = 0.0002 (95% CI: -0.0004, 0.0007, p = 0.58) C-index reference model = 0.69 (95% CI: 0.66, 0.72)  C-index reference model + HDP = 0.69 (95% CI: 0.66, 0.72) C-index difference = 0.00014 (95% CI: -0.001, 0.002) | | | | | | | |

| **Table S6.** Risk reclassification for 10-year CVD prediction in women age 60 years with history of hypertensive disorders of pregnancy added to the reference model | | | | | | | |
| --- | --- | --- | --- | --- | --- | --- | --- |
| **Women with CVD events during 10-year follow-up** | | | | | | | |
|  | **Reference model + history of HDP** | | | | |  | |
| **Reference model** | **0 to <5%** | | **5 to <10%** | | **≥10%** | **Total** | |
| **0 to <5%** | 50 (92.6) | | 4 (7.4) | | 0 | 54 (13.3) | |
| **5 to <10%** | 0 | | 204 (98.6) | | 3 (1.5) | 207 (51.1) | |
| **≥10%** | 0 | | 3 (2.1) | | 141 (97.9) | 144 (35.6) | |
| **Total** | 50 (12.4) | | 211 (52.1) | | 144 (35.6) | 405 | |
| **Women with no CVD events during 10-year follow-up** | | | | | | | |
|  | | **Reference model + history of HDP** | | | | | |
| **Reference model** | | **0 to <5%** | | **5 to <10%** | **≥10%** | | **Total** |
| **0 to <5%** | 1,144 (97.8) | | 26 (2.2) | | 0 | 1,170 (24.6) | |
| **5 to <10%** | 27 (0.99) | | 2,662 (98.0) | | 28 (1.0) | 2,717 (57.2) | |
| **≥10%** | 0 | | 28 (3.2) | | 836 (96.8) | 864 (18.2) | |
| **Total** | 1,171 (24.7) | | 2,716 (57.2) | | 864 (18.2) | 4,751 | |
| CI: Confidence interval; CVD: Cardiovascular disease; HDP: Hypertensive disorders of pregnancy; IDI: Integrated discriminatory improvement; NRI: Net reclassification improvement  Data presented as n (percentage)  Women censored due to non-events within ten years of baseline are excluded from the table (n=204)  Categorical NRI for events = 0.010 (95% CI: -0.005, 0.026, p = 0.20) Categorical NRI for non-events = 0.001 (95% CI: -0.004, 0.005, p = 0.78)  IDI = 0.0002 (95% CI: 0.00001, 0.0004, p = 0.05) C-index reference model = 0.63 (95% CI: 0.61, 0.66)  C-index reference model + HDP = 0.63 (95% CI: 0.61, 0.66) C-index difference = 0.0003 (95% CI: -0.002, 0.003) | | | | | | | |

| **Table S7.** Risk reclassification for 10-year CVD prediction with low birth weight offspring added to the reference model not stratified by age at baseline | | | | | | | |
| --- | --- | --- | --- | --- | --- | --- | --- |
| **Women with CVD events during 10-year follow-up** | | | | | | | |
|  | **Reference model + LBW offspring (<2,500 g)** | | | | | | |
| **Reference model** | **0 to <5%** | | **5 to <10%** | | **≥10%** | **Total** | |
| **0 to <5%** | 176 (95.1) | | 9 (4.9) | | 0 | 185 (30.0) | |
| **5 to <10%** | 7 (2.8) | | 235 (94.4) | | 7 (2.8) | 249 (40.4) | |
| **≥10%** | 0 | | 3 (1.7) | | 179 (98.4) | 182 (29.6) | |
| **Total** | 183 (29.7) | | 247 (40.1) | | 186 (30.2) | 616 | |
| **Women with no CVD events during 10-year follow-up** | | | | | | | |
|  | | **Reference model + LBW offspring (<2,500 g)** | | | | | |
| **Reference model** | | **0 to <5%** | | **5 to <10%** | **≥10%** | | **Total** |
| **0 to <5%** | 5,769 (97.5) | | 147 (2.5) | | 0 | 5,916 (58.3) | |
| **5 to <10%** | 142 (4.5) | | 2,984 (93.9) | | 53 (1.7) | 3,179 (31.3) | |
| **≥10%** | 0 | | 33 (3.1) | | 1,017 (96.9) | 1,050 (10.4) | |
| **Total** | 5,911 (58.3) | | 3,164 (31.2) | | 1,070 (10.6) | 10,145 | |
| CI: Confidence interval; CVD: Cardiovascular disease; NRI: Net reclassification improvement; IDI: Integrated discriminatory improvement; LBW: Low birth weight (<2,500 g)  Data presented as n (percentage)  Women censored due to non-events within ten years of baseline are excluded from table (n=349). Model includes interaction term between age at baseline and LBW offspring.  Categorical NRI for events = 0.010 (95% CI: -0.006, 0.026, p = 0.22) Categorical NRI for non-events = -0.002 (95% CI: -0.006, 0.002, p = 0.24)  IDI = 0.0003 (95% CI: -0.00004, 0.00076, p = 0.12)  C-index reference model = 0.69 (95% CI: 0.67, 0.71)  C-index reference model + LBW offspring = 0.69 (95% CI: 0.67, 0.71) C-index difference with LBW = 0.002 (95% CI: -0.001, 0.006) | | | | | | | |

| **Table S8.** Risk reclassification for 10-year CVD prediction with history of hypertensive disorders of pregnancy added to the reference model not stratified by age at baseline | | | | | | |
| --- | --- | --- | --- | --- | --- | --- |
| **Women with CVD events during 10-year follow-up** | | | | | | |
|  | **Reference model + history of HDP** | | | | |  |
| **Reference model** | **0 to <5%** | | **5 to <10%** | | **≥10%** | **Total** |
| **0 to <5%** | 180 (97.3) | | 5 (2.7) | | 0 | 185 (30.0) |
| **5 to <10%** | 3 (1.2) | | 239 (96.0) | | 7 (2.8) | 249 (40.4) |
| **≥10%** | 0 | | 6 (3.3) | | 176 (96.7) | 182 (29.6) |
| **Total** | 183 (29.7) | | 250 (40.6) | | 183 (29.7) | 616 |
| **Women with no CVD events during 10-year follow-up** | | | | | | |
|  | | **Reference model + history of HDP** | | | | |
| **Reference model** | | **0 to <5%** | | **5 to <10%** | **≥10%** | **Total** |
| **0 to <5%** | 5,840 (98.7) | | 76 (1.3) | | 0 | 5,916 (58.3) |
| **5 to <10%** | 68 (2.1) | | 3,074 (96.7) | | 37 (1.2) | 3,179 (31.3) |
| **≥10%** | 0 | | 40 (3.8) | | 1,010 (96.2) | 1,050 (10.4) |
| **Total** | 5,908 (58.2) | | 3,190 (31.4) | | 1,047 (10.3) | 10,145 |
| CI: Confidence interval; CVD: Cardiovascular disease; HDP: Hypertensive disorders of pregnancy; IDI: Integrated discriminatory improvement; NRI: Net reclassification improvement  Data presented as n (percentage)  Women censored due to non-events within ten years of baseline are excluded from the table (n=349). Model includes interaction term between age at baseline and history of HDP.  Categorical NRI for events = 0.005 (95% CI: -0.009, 0.020, p = 0.48) Categorical NRI non-events = -0.0006 (95% CI: -0.003, 0.002, p = 0.66)  IDI = 0.0002 (95% CI: -0.0002, 0.0006, p = 0.24)  C-index reference model = 0.69 (95% CI: 0.67, 0.71) C-index reference model + HDP = 0.69 (95% CI: 0.67, 0.71) C-index difference = 0.0004 (95% CI: -0.001, 0.002) | | | | | | |

**References**

1. Sandström A, Nyström L. Uppbyggnad av ett medicinskt födelseregister för perioden 1955–1972. Socialmedicinsk Tidskr. 1985:95 – 7.

2. Mogren I, Hogberg U, Winkvist A, Stenlund H. Familial occurrence of preeclampsia. Epidemiology. 1999;10:518–22.

3. Mogren I, Hogberg U, Stegmayr B, Lindahl B, Stenlund H. Fetal exposure, heredity and risk indicators for cardiovascular disease in a Swedish welfare cohort. Int J Epidemiol. 2001;30:853–62.

4. Mogren I, Damber L, Tavelin B, Högberg U. Characteristics of pregnancy and birth and malignancy in the offspring (Sweden). Cancer Causes Control. 1999;10:85–94.

5. Ludvigsson JF, Otterblad-Olausson P, Pettersson BU, Ekbom A. The Swedish personal identity number: possibilities and pitfalls in healthcare and medical research. Eur. J. Epidemiol. 2009;24:659–67.

6. Ros HS, Cnattingius S, Lipworth L. Comparison of Risk Factors for Preeclampsia and Gestational Hypertension in a Population-based Cohort Study. Am. J. Epidemiol. 1998;147:1062–70.

7. Salonen Ros H, Lichtenstein P, Lipworth L, Cnattingius S. Genetic effects on the liability of developing pre-eclampsia and gestational hypertension. Am. J. Med. Genet. 2000;91:256–60.

8. Thomsen LCV, Klungsøyr K, Roten LT, Tappert C, Araya E, Bærheim G, et al. Validity of the diagnosis of pre-eclampsia in the Medical Birth Registry of Norway. Acta Obstet. Gynecol. Scand. 2013;92:943–50.

9. Stone NJ, Robinson JG, Lichtenstein AH, Bairey Merz CN, Blum CB, Eckel RH, et al. 2013 ACC/AHA Guideline on the Treatment of Blood Cholesterol to Reduce Atherosclerotic Cardiovascular Risk in Adults: A Report of the American College of Cardiology/American Heart Association Task Force on Practice Guidelines. J. Am. Coll. Cardiol. 2014;63:2889–934.
